# Glyoxalase I Gene Deletion Mutants of *Leishmania donovani* Exhibit Reduced Methylglyoxal Detoxification

**DOI:** 10.1371/journal.pone.0006805

**Published:** 2009-08-27

**Authors:** Swati C. Chauhan, Rentala Madhubala

**Affiliations:** School of Life Sciences, Jawaharlal Nehru University, New Delhi, India; INSERM U567, Institut Cochin, France

## Abstract

**Background:**

Glyoxalase I is a metalloenzyme of the glyoxalase pathway that plays a central role in eliminating the toxic metabolite methyglyoxal. The protozoan parasite *Leishmania donovani* possesses a unique trypanothione dependent glyoxalase system.

**Principal Findings:**

Analysis of the *L. donovani GLOI* sequence predicted a mitochondrial targeting sequence, suggesting that the enzyme is likely to be targeted to the mitochondria. In order to determine definitively the intracellular localization of GLOI in *L. donovani*, a full-length *GLOI* gene was fused to green fluorescent protein (GFP) gene to generate a chimeric construct. Confocal microscopy of *L. donovani* promastigotes carrying this chimeric construct and immunofluorescence microscopy using anti-GLOI antibodies demonstrated that GLOI is localized in the kinetoplast of the parasite apart from the cytosol. To study the physiological role of *GLOI* in *Leishmania,* we first created promastigote mutants heterozygous for *GLOI* by targeted gene replacement using either hygromycin or neomycin phosphotransferases as selectable markers. Heterozygous mutants of *L. donovani* display a slower growth rate, have lower glyoxalase I activity and have reduced ability to detoxify methylglyoxal in comparison to the wild-type parasites. Complementation of the heterozygous mutant with an episomal *GLOI* construct showed the restoration of heterozygous mutant phenotype nearly fully to that of the wild-type. Null mutants were obtained only after *GLOI* was expressed from an episome in heterozygous mutants.

**Conclusions:**

We for the first time report localization of GLOI in *L. donovani* in the kinetoplast. To study the physiological role of GLOI in *Leishmania*, we have generated GLOI attenuated strains by targeted gene replacement and report that GLOI is likely to be an important gene since *GLOI* mutants in *L. donovani* showed altered phenotype. The present data supports that the GLOI plays an essential role in the survival of this pathogenic organism and that inhibition of the enzyme potentiates the toxicity of methylglyoxal.

## Introduction

Leishmaniasis constitutes a wide spectrum of diseases ranging from the simple self-limiting cutaneous form to the debilitating visceral form, which is often fatal if left untreated. The protozoan parasite *Leishmania donovani* is the major causative agent of visceral leishmaniasis. The current challenges in its chemotherapy include widespread resistance to pentavalent antimony; absence of safe and cost-effective antileishmanial agents and relapses in HIV *Leishmania* co-infected patients. Thus an urgent need exists to look for newer and more effective drug targets to treat leishmaniasis.

The glyoxalase system of *Leishmania* is one such pathway that may be exploited successfully for the development of anti-parasitic drugs. The glyoxalase system is a ubiquitous thiol-dependent detoxification pathway [1]. This system comprises of two enzymes, glyoxalase I (GLOI) (lactoylglutathione lyase, EC 4.4.1.5) and glyoxalase II (GLOII) (hydroxyacylglutathione hydrolase, EC 3.1.2.6). Glyoxalase I catalyses the formation of S-D lactoyl glutathione from the hemithioacetal formed nonenzymatically from methylglyoxal and glutathione. Glyoxalase II converts S-D lactoyl glutathione to lactate and free glutathione [2]. Recent reports demonstrated a unique glyoxalase system in pathogenic kinetoplastids, as a consequence of this protozoan possessing an unusual thiol metabolism [3], [4], [5]. Glyoxalase I and glyoxalase II in kinetoplastids showed high level of specificity to trypanothine hemithioacetal and little activity with glutathione hemithioacetal [3], [4], [5], [6]. The unique nature of this trypanothione-dependent glyoxalase pathway represents a novel chemotherapeutic target for the kinetoplastids. A major function of the glyoxalase pathway is believed to be detoxification of α-ketoaldehydes, especially methylglyoxal (MG). MG is a cytotoxic metabolite produced primarily as a by-product of glycolysis through nonenzymatic phosphate elimination from the glycolytic pathway intermediates, dihydroxyacetone phosphate and glyceraldehyde 3-phosphate [2]. The glyoxalase enzymes have been reported to have a regulatory role in cell division including cell proliferation, tissue regeneration, and malignancy besides detoxification of methylglyoxal [7]. We had earlier cloned and characterized the genes encoding glyoxalase I (*GLOI*) (AY739896) and glyoxalase II (*GLOII*) (AY851655) from *Leishmania donovani* [5], [6]. Glyoxalase I is a single copy gene and is placed at a single chromosomal band of ∼2.2 Mb. Multiple sequence alignment and homology modeling clearly showed that there is a crucial difference in the active site of human and *Leishmania* glyoxalase I enzymes suggesting that the glyoxalase I may be a potential target for drug design [5].

In the present study we report that *L. donovani* promastigotes carrying a GLOI-green fluorescent protein (GLOI-GFP) chimeric construct display localization of GLOI in the kinetoplast of the parasite apart from the cytosol. To study the physiological role of GLOI in *Leishmania*, we have generated GLOI attenuated strains by targeted gene replacement. Attempts to disrupt both alleles of *GLOI* in *L. donovani* promastigotes resulted in change in levels of cell ploidy. These GLOI mutants were phenotypically characterized and showed reduced growth, lower GLOI enzymatic activity and had reduced ability to detoxify methylglyoxal in comparison to the wild-type parasites. Null mutants of *GLOI* were obtained only after rescuing the heterozygous mutants with an episomal copy of *GLOI.* This data suggests that the GLOI is essential to *L. donovani*.

## Results

### Subcellular localization of Glyoxalase I in L. donovani

**Figure 1:**
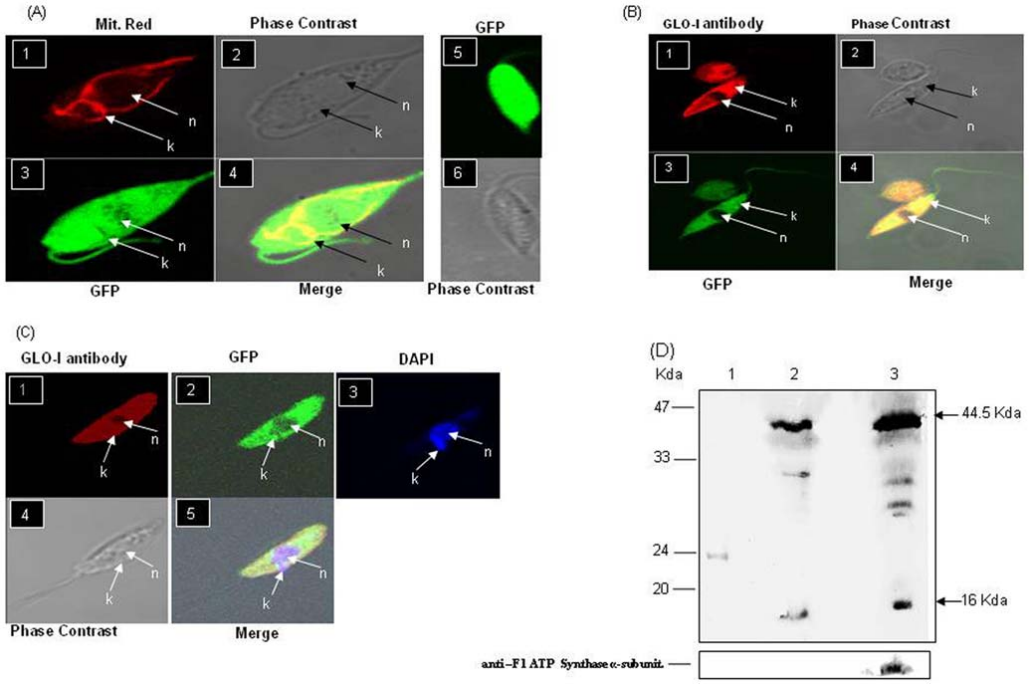
Localization of GLOI in *L. donovani*. A: Panel 1: confocal microscopy of wild type *L. donovani* transfected with pGEM7zf-αNEOα-GFP-GLOI expressing GLOI as a GFP translational fusion protein and stained with Mitotracker-Red CMX Ros dye, Panel 2: phase contrast image, Panel 3: wild type *L. donovani* expressing GLOI as a GFP translational fusion protein, Panel 4: merged micrograph, Panel 5: wild type *L. donovani* transfected with pGEM7zf-αNEOα-GFP vector alone expressing GFP, Panel 6: phase contrast image B: Panel 1: Immunoflourescence analysis by confocal micrograph of wild type *L. donovani* parasite transfected with pGEM7zf-αNEOα-GFP-GLOI,using anti–GLOI antibody, Panel 2: phase contrast image. Panel 3: wild type *L. donovani* expressing GLOI as a GFP translational fusion protein, 4: merged micrograph C: Panel 1: Immunoflourescence analysis by confocal micrograph of wild type *L. donovani* parasite transfected with pGEM7zf-αNEOα-GFP-GLOI using anti-GLOI antibody, Panel 2: wild type *L. donovani* expressing GLOI as a GFP translational fusion protein, Panel 3: stained with DAPI, Panel 4: phase contrast image, Panel 5: merged micrograph. D: Western blotting using anti-GLOI antibody. Cytosolic and mitochondrial fractions were separated from late log phase promastigotes (1×10^8^) expressing GLOI as a GFP translational fusion protein. Lane 1: recombinant GLOI, Lane 2: cytosolic fraction, Lane 3: mitochondrial fraction. The blot was also probed with mitochondrial specific anti-F1 ATP synthase α-subunit. Results are representative data from three separate experiments.

Amino acid sequence analysis of the LdGLOI protein in-silico using pSORT [8] and MitoProt II [9] web-based programs predicted that GLOI is localized to the mitochondria with a *P* = 0.8780 probability. However, no cleavage site was predicted for the mitochondrial targeting sequence using Target P web based program [10]. To determine the localization of GLOI in intracellular organelles, we investigated the localization of a GFP-tagged version of GLOI in *L. donovani* and compared with the wild type parasites, transfected with the vector alone (pGEM-7zfαNeoα-GFP) (Fig. 1A, panel 5 and 6). Confocal microscopy showed that the GLOI−GFP fusion protein was localized both in the cytosol and the *Leishmania* kinetoplast (Fig. 1A,), as seen by the clear co-localization of the GLOI−GFP with the fluorescence associated to the MitoTracker Red CMX Ros that reveals the position of mitochondria within the promastigotes (Fig. 1A, panel 1, 3 and 4). Immuno-localization of GLOI using anti-GLOI antibodies and Alexa 546 labeled anti mouse IgG as secondary antibody in the *L.donovani* expressing GLOI as GFP translational fusion protein, further confirmed the presence of GLOI in the kinetoplast and the cytosol and not in the nucleus (Fig. 1B panel 1, 3 and 4). Immuno-localization of GLOI using anti-GLOI antibodies and Alexa 546 labeled anti mouse IgG as secondary antibody was also done and counter stained with DAPI to confirm the position of the nucleus and the kinetoplast with in the parasites (Fig. 1C, panel 1, 2, 3 and 5). Western blot analysis with both the cytosolic and mitochondrial fraction (100 µg protein lysate) of the GLOI-GFP transfected parasites showed a band of ∼44.5 kDa that corresponded to the size of the fusion protein and a band of 16 kDa that corresponded to the native *L.donovani* GLOI protein. These experiments confirmed the presence of GLOI with in the kinetoplast and the cytosol of the parasites (Fig. 1D).The mitochondrial fraction was further confirmed by reprobing the blot with mitochondrial specific anti-F1 ATP synthase α-subunit (Fig. 1D).

### Construction and Molecular characterization of GLOI mutants

**Figure 2:**
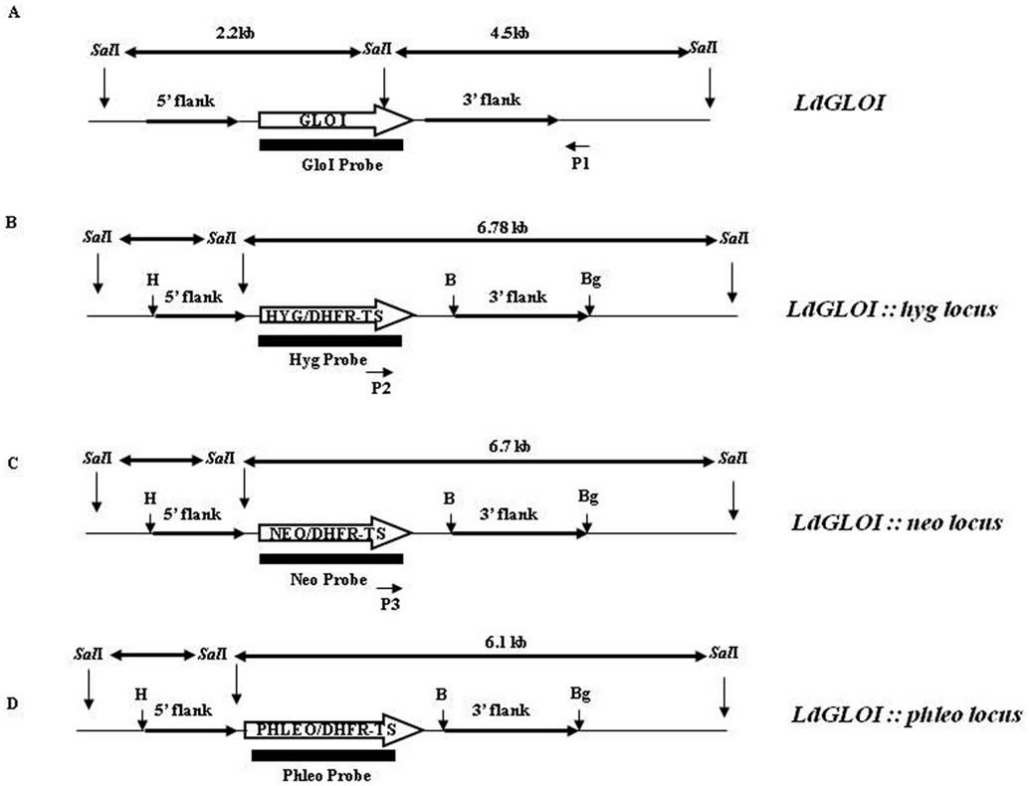
Schematic representation of the wild type and the *hyg*, *neo and phleo* targeted *Ld GLOI* locus. *Sal*I restriction sites were used to analyze the recombination events. The restriction fragments obtained after *Sal* I digestion are indicated by double headed arrows along with their respective size. Thick arrows indicate the ORF of genes as indicated. Bold arrows indicate the 5′ and 3′ flanking regions of *GLOI* ORF. Solid bars indicate the probes used for Southern blot analysis. Primers P 1 and P 2 were used to check the correct integration of *hyg* cassette and primers P 1 and P 3 were used to check the correct integration of *neo* cassette. Restriction sites (H- *Hin*dIII, B- *Bam*HI, Bg - *Bgl*II, *Sal*I) used for cloning 5′ and 3′ flank are also indicated.

**Figure 3:**
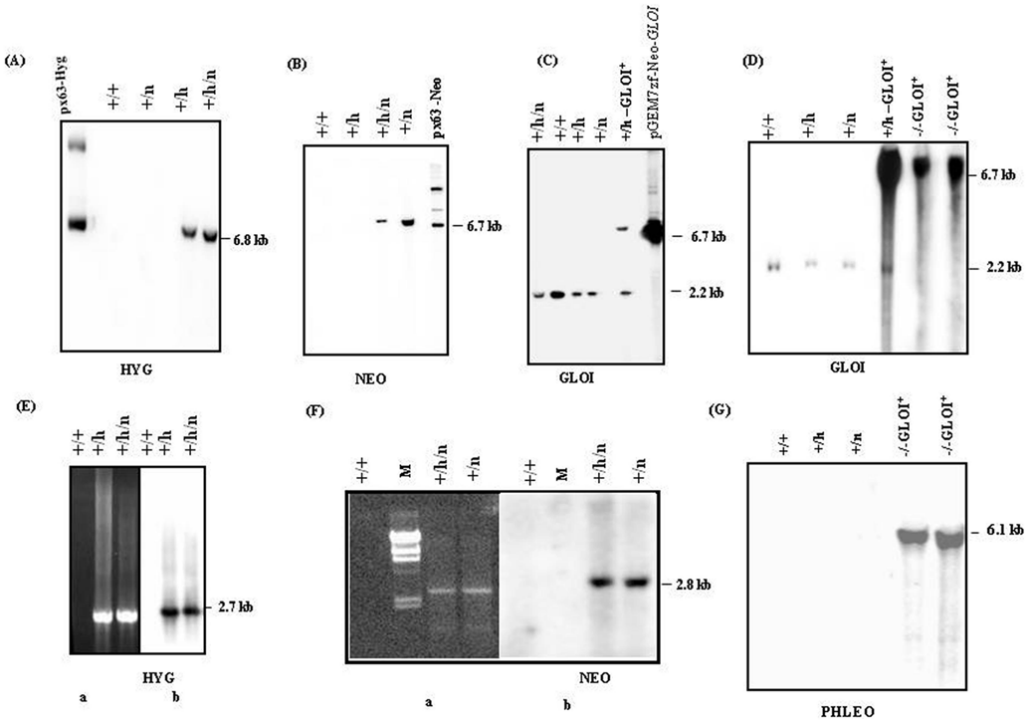
Southern blot and PCR analysis of *GLOI* knockout mutants. Southern blot analysis of wild type and mutant parasites to confirm the recombination events. Equal amount of genomic DNA (5 µg) was digested with *Sal*I restriction enzyme and separated on a 0.8% agarose gel. The DNA was blotted on to membrane and hybridized with *hyg* specific probe (A), *NEO* specific probe (B), *GLOI* specific probe (C) and *PHLEO* specific probe (D). (+/+) represents Wild type; (+/h) represents heterozygote *LdGLOI::HYG* clone; (+/n) represents heterozygote *LdGLOI :: NEO* clone; (+/h/n) represents *L. donovani* double targeted transfectant, *LdGLOI :: HYG :: NEO* clone, (+/h-GLOI^+^) represents *GLOI* complementation mutant and (−/− GLOI^+^) represents *GLOI* null mutants rescued with an episomal copy of *GLOI.* M represents DNA molecular weight marker; uncut pX63-HYG and pX63-NEO and linearized pGEM7zfαNeoα-GLOI^+^ plasmids were used as positive controls for the respective blots probed with *HYG, NEO* and *GLOI*. PCR was carried out with primers P1 and P2 for checking *hyg* cassette integration (E, panel a) and with primers P1 and P3 for checking *neo* cassette integration (F, panel a). Southern blot analysis of the corresponding PCR gels probed with *HYG* (E, panel b) and *NEO* (F, panel b). G: Southern blot analysis of genomic DNA digested with *Sal*I and probed with PHLEO probe to verify *phleo* cassette integration.

From our earlier studies it is known that *GLOI* is a single copy gene in *L. donovani* and is placed at a single chromosomal band of ∼2.2 Mb [5]. Since *Leishmania* is considered to be diploid and no sexual crosses have been achieved, homologous recombination strategy has been used in an attempt to generate transgenic parasites lacking both alleles of *GLOI*. The wild type parasites were transfected individually with the *hyg* and *neo* replacement cassettes to create the *GLOI* heterozygotes; *Ld GLOI :: HYG* (+/h) and *Ld GLOI :: NEO* (+/n) respectively (Fig. 2). The Hyg and Neo transfectants were selected against hygromycin (200 µg/ml) and G-418 (40 µg/ml) respectively. The representative clone from the *Ld GLOI :: HYG* (+/h) and *Ld GLOI :: NEO* (+/n) single knock out mutants were screened for the correct integration of the *hyg* and *neo* replacement cassette respectively. The *Sal*I digested genomic DNA from the wild-type *L. donovani* and representative *L. donovani* heterozygous clones *Ld GLOI :: HYG* (+/h) and *LdGLOI :: NEO* (+/n) were subjected to Southern blot analysis and results are presented in Fig. 3. The integrations of the selectable markers were confirmed by hybridization with *hyg* and *neo* specific probes. The *hyg* specific probe (1026-bp) resulted in no hybridizing bands with *Sal*I-digested genomic DNA from wild type parasites and *Ld GLOI::NEO* (+/n) clones, while a single band of ∼6.8 kb, corresponding to the *hyg* based replacement fragment was detected in *L. donovani* heterozygous clones, *Ld GLOI :: HYG* (+/h) (Fig. 3A). The *neo* specific probe (800-bp) resulted in no hybridizing band with *Sal*I digested genomic DNA from the wild-type (+/+) parasites and representative *Ld GLOI :: HYG* (+/h) clones but a single band of ∼6.7 kb was detected in the *Ld GLOI :: NEO* clone (+/n) (Fig. 3B). Southern blot analysis of *Sal*I-digested genomic DNA from wild-type parasites (+/+), *L. donovani* heterozygote*s Ld GLOI :: HYG* (+/h), *Ld GLO :: NEO* (+/n), a double transfectant mutant clone *Ld GLOI :: HYG :: NEO* (+/h/n) and a *GLOI* complementation mutant (+/h–GLOI^+^) was performed and blots were hybridized with the *GLOI* ORF probe (426 bp). An expected 2.2 kb band was recognized in the DNA from wild-type *L. donovani* (+/+) and *L. donovani* heterozygous mutants *Ld GLOI :: HYG* clone (+/h), *Ld GLOI::NEO* clone (+/n) and GLOI complementation mutant (+/h –GLOI^+^). An additional and expected band of 6.7 Kb was observed in the (+/h)-*GLOI* ^+^ transfectant that corresponds to the presence of an episomal copy of *GLOI* in the *Ld GLOI :: HYG* (+/h) background. However, the double transfectant mutant clone, *Ld GLOI :: HYG :: NEO* (+/h/n) showed the presence of a *GLOI* specific band of 2.2 kb (Fig. 3C). A total of ten double transfectant mutant (+/h/n) clones were screened and a 2.2 kb specific *GLOI* band was present in all the clones (representative data from one clone is shown). Although *GLOI* gene disruption by *neo* and *hyg* took place, as clearly shown from the hybridization studies with the *hyg* (Fig. 3A) and *neo* probes (Fig. 3B), one *GLOI* allele remained intact since the 2.2 kb genomic *Sal*I fragment was still present in the double targeted mutants (Fig. 3C, lane: +/h/n). The gene replacement of four of the (+h/n) clones was further evaluated by polymerase chain reaction (PCR).

PCR based approach was also used to analyze for the correct integration of the *hyg* and *neo* based replacement constructs. Integration specific primers P1 and P2 as mentioned in the material and methods and shown in Fig. 2 were used to determine *hyg* specific integration (Fig. 3E), whereas P1 and P3 was used to verify *neo* specific integration (Fig. 3F). Southern blotting of the PCR products of the wild type (+/+), *Ld GLOI :: HYG* clone (+/h), *Ld GLOI :: NEO* clone (+/n) and double transfectant mutant clone *Ld GLOI::HYG::NEO* (+/h/n) using *hyg* (Fig. 3E, panel b) and *neo* (Fig. 3F, panel b) specific probes further confirmed the expected integration. These observations confirmed the generation of single knockouts of *GLOI* by both *hyg* and *neo* based replacement cassettes. PCR studies further confirmed that in the double transfectant mutant clone *Ld GLOI :: HYG :: NEO* (+/h/n) disruption of *GLOI* gene by *neo* (Fig. 3F, panel b) and *hyg* (Fig. 3E, panel b) took place.

**Figure 4:**
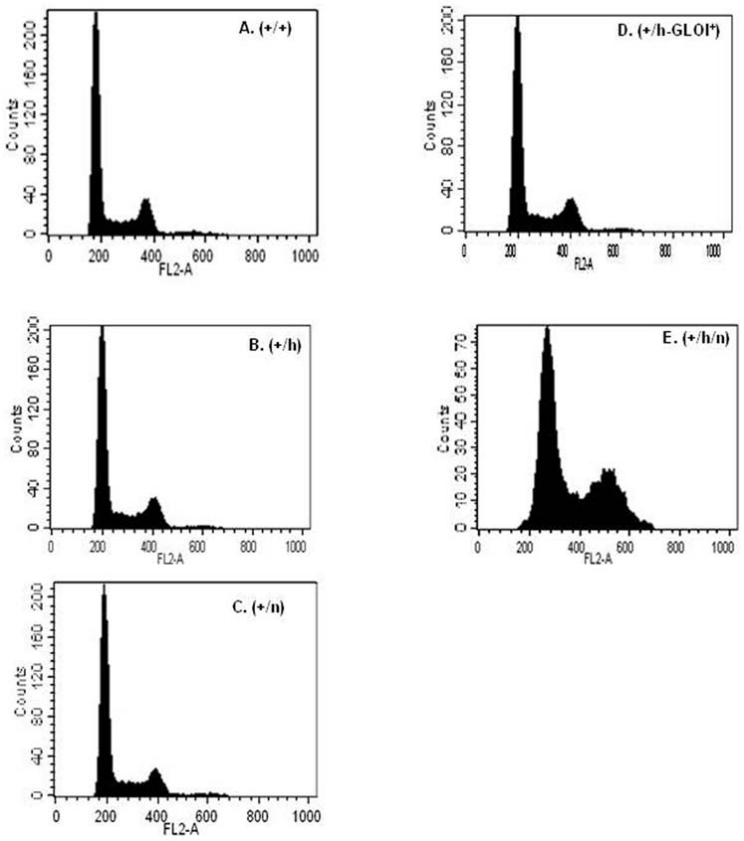
DNA content analysis. Wild type and GLOI mutant *L.donovani* cell lines were analysed for DNA content using flow cytometry. A: Wild type (+/+): B: heterozygote *LdGLOI :: HYG* clone (+/h); C: heterozygote *LdGLOI :: NEO* clone (+/n);, D: *GLOI* complementation mutant (+/h-GLOI^+^); E: *L. donovani* double targeted transfectant, *LdGLOI :: HYG :: NEO* clone (+/h/n) were fixed and stained with propidium iodide and analysed using a Beckton Dickinson flow cytometer(FACScalibur). Representative peaks of G1 and G2 phase are shown as predicted by the CELL QUEST software. Results are representative data from three separate experiments.

Since the double transfectant mutant clones *Ld GLOI::HYG::NEO* (+/h/n) were found to contain 2.2-kb wild type *GLOI* allele as well as *hyg* and *neo* integrations, DNA content based analysis was performed to check the ploidy levels of the cells. This was determined by fluorescence activated cell sorting (FACS) analysis of fixed cells stained with propidium iodide (Fig. 4). In the wild type cells (+/+), two peaks corresponding to 2N (G1) and 4N (G2) DNA content were observed as expected (Fig. 4 panel A). Similar pattern was observed with the heterozygote mutants (+/h) and (+/n) and GLOI complementation mutant (+/h-GLOI^+^) (Fig. 4. panels B, C and D) thereby showing that these clones contain normal diploid DNA content similar to the wild type cells. However, analysis of the double transfected mutant clone (+/h/n) showed peaks that corresponded to 3N and ∼6N DNA content (Fig. 4 panel E) (representative data from one of the clones is shown). This suggests a difference in the ploidy level in the cell population of the double transfectants when compared to the wild type cells. The G1 peak of the double transfected mutant clone indicates that these cells are aneuploid (Fig. 4. panel E).

In order to create null mutant of *GLOI*, a further attempt was made to express the gene from an episome prior to disrupting the second *GLOI* allele. The heterozygous GLOI mutant with an episomal copy of GLOI (+/h-GLOI^+^) was transfected with the linearized pX63-PHLEO-Δ*GLOI* construct. The transfectants were selected against hygromycin (200 µg/ml), G418 (40 µg/ml) and phleomycin (25 µg/ml). The null mutants thus obtained were designated as (−/−GLOI^+^). The *Sal*I digested genomic DNA from nine clones was subjected to Southern blotting and the blot was hybridized with *GLOI* (426-bp) (Fig. 3D) or *phleo* (375-bp) specific probe (Fig. 3G). A representative data from two (−/−GLOI^+^) clones is shown in Fig. 3D and 3G. The 2.2 kb *GLOI* fragment was present in the wild type *L. donovani* (+/+), heterozygous mutants, *Ld GLOI :: HYG* clone (+/h), *LdGLOI::NEO* clone (+/n) and GLOI complementation mutant (+/h –GLOI^+^). The two mutant clones (−/−GLOI^+^) lacked 2.2-kb wild type *GLOI* fragment. These two clones were found to contain an intensely hybridizing fragment of 6.7-kb which corresponded to the presence of an episomal copy of *GLOI* in the *Ld GLOI :: HYG* (+/h) background. These clones were also checked for the correct integration of the *phleo* replacement cassette by Southern blot analysis of *Sal*I digested genomic DNA followed by hybridization with the *phleo* probe. Fig. 3G shows that both the clones (−/−GLOI^+^) contain 6.1-kb fragment indicative of the correct integration of the *phleo* cassette.

A total of at least two clones of each of the mutant lines were picked and screened for phenotypic characterization. Representative data from one clone from each mutant line is presented below.

### GLOI heterozygous mutants are growth defective

**Figure 5:**
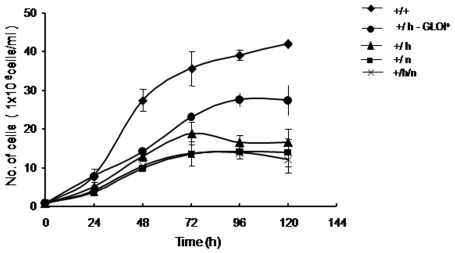
Growth profile of promastigotes of wild type (+/+), heterozygote *LdGLOI :: HYG* clone (+/h); heterozygote *LdGLOI :: NEO* clone (+/n); *L. donovani* double targeted transfectant, *LdGLOI :: HYG :: NEO* clone (+/h/n), *GLOI* complementation mutant (+/h-GLOI^+^). Growth comparison was done by inoculating stationary phase cells in modified M199 medium with 10% FBS at a density of 1×10^6^ cells/ml. Cells were counted at 24 h intervals using a Neubauer hemocytometer. Results are representative data from two separate experiments with mean±SD of triplicates in each set.

In order to verify phenotypic alteration in these gene replacement mutants, the growth rate of each of the cell lines was determined for a period of 120 h at an interval of 24 h. Fig. 5 shows the growth of the wild-type *L. donovani*, *Ld GLOI :: HYG* (+/h), *Ld GLOI :: NEO* (+/n) and complementation clone (+/h)-*GLOI* ^+^ and double targeted transfectant clone *Ld GLOI :: HYG :: NEO* (+/h/n). The promastigotes of the *GLOI* heterozygous mutants (+/h) and (+/n) showed a slower growth rate when compared to that of the wild-type cells (+/+). The *GLOI* heterozygous mutants (+/h) clone when complemented with an episomal copy of *GLOI* showed an increase in the growth rate as compared to the heterozygous mutant, thereby revealing that the presence of *GLOI* as an episomal copy facilitates in overcoming the defect in the growth that probably has arisen because of the deletion of one of the wild type *GLOI* allele. Double targeted transfectant clone (+/h/n) had growth pattern similar to that of the heterozygous GLOI mutant strains.

### GLO I heterozygous mutants exhibit reduced methylglyoxal (MG) detoxification

The effective concentration of methylglyoxal (MG) that inhibited cell growth by 50% (IC_50_) was 1.0±0.1 mM in the wild-type *L. donovani*. However, that of the *L. donovani* heterozygous mutants, *Ld GLOI :: HYG* (+/h) and *Ld GLOI :: NEO* (+/n), was 0.45±0.03 mM and 0.30±0.02 mM. In the case of the double targeted transfectant clone (+/h/n) the effective concentration was 0.41±0.04 (Table 1). This data indicates that *L. donovani* mutants probably have a significantly reduced rate of metabolism of MG as compared to the wild-type parasites suggesting that the glyoxalase pathway is the dominant route for detoxification of MG in *L. donovani*. However, the heterozygous mutant clone *Ld GLOI :: HYG* (+/h) when transfected with an episomal copy of *GLOI* (+/h-*GLOI* ^+^) had an IC_50_ = 0.8±0.07 mM towards MG. This date indicates that complementation of *GLOI* in heterozygous mutant clone *Ld GLOI :: HYG* (+/h) decreases the sensitivity towards methylglyoxal.

**Table 1:** Effect of methyglyoxal on the cell viability of the Wild type and the mutant parasites.

Strain	IC_50_±S.D. (mM)
Wild type (+/+)	1±0.14
*Ld GLOI :: hyg* (+/h)	0.45±0.03*
*Ld GLOI :: neo* (+/n)	0.3±0.02*
*Ld GLOI hyg :: neo* (+/h/n)	0.41±0.04*
*Ld GLOI :: hyg GLO I* ^+^(+/h -*GLOI* ^+^)	0.8±0.07

IC_50_ represents the concentration of methylglyoxal, which inhibited the growth of the wild type and the mutant parasites by 50%. Each value is a mean±S.D. of three independent experiments.

* Significantly different from the wild type (P<0.001).

### Reduction in glyoxalase I activity in GLOI heterozygous mutant

The specific activity of GLOI in heterozygous mutants, *Ld GLOI :: HYG* (+/h) and *Ld GLOI :: NEO* (+/n), was 173±31 nmol min^−1^ mg^−1^protein and 116.5±33 nmol min^−1^ mg^−1^protein respectively. The specific activity in the double targeted transfectant clone (+/h/n) was 109.5±13 nmol min^−1^ mg^−1^protein. However, a higher specific activity of GLOI in the wild-type *L. donovani* (486±19.8 nmol min^−1^ mg^−1^protein) was observed when compared to the respective mutants. Thus, Glyoxalase I activity in *L. donovani* mutants showed an approximately two fold reduction as compared to the wild-type *L. donovani.* The heterozygous mutant *Ld GLOI:: HYG* (+/h) when complemented with an episomal copy of GLOI had a specific activity of 345±28 nmol min^−1^ mg^−1^protein (Table 2).

**Table 2:** Comparison of the Glyoxalase I activity of the Wild type and the mutant strains of glyoxalase I.

Strain	Specific activity±S.D (nmoles/min/mg)
Wild type (+/+)	486±19.8
*Ld GLOI :: hyg* (+/h)	173±31*
*Ld GLOI :: neo* (+/n)	116.5±33*
*Ld GLOI hyg :: neo* (+/h/n)	109.5±13*
*Ld GLOI :: hyg GLO I* ^+^(+/h -*GLOI* ^+^)	345±28

The Glyoxalase I activity of the wild type and mutant parasites was determined. Results are mean±S.D. values of three independent experiments. *Significantly different from the wild type (P<0.001).

The rescued null mutant clones (−/−GLOI^+^) had normal diploid content. The growth pattern, IC_50_ towards MG and specific activity of GLOI null mutant clones (−/−GLOI^+^) was similar to that of the GLOI complementation mutant (+/h –GLOI^+^) (data not shown).

## Discussion

The glyoxalase system, consisting of the enzymes glyoxalase I (GLOI) and glyoxalase II (GLOII), is an integral component of cellular metabolism in mammalian systems. Although the glyoxalase pathway was reported as early as 1913 [11], [12] and is widely distributed in living organisms, its full biological function has never been elucidated. A major function of the glyoxalase pathway is believed to be detoxification of α-ketoaldehydes, especially methylglyoxal (MG). MG is a cytotoxic metabolite produced primarily as a by-product of glycolysis [13]. Another role of GLOI and methylglyoxal is their involvement in the regulation of cellular growth [14]. In tumor tissues high activities of glyoxalase I have also been reported [15]. Inhibitors of glyoxalase I have been reported to be selectively toxic to proliferating cells, this could be due to increased accumulation of methylglyoxal that could lead to inhibition of DNA synthesis [16], [17]. Glyoxalase I inhibitors have been reported to have antimalarial [18] and antitrypanosomal activities [19]. The glyoxalase system of the pathogenic kinetoplastids has been recently reported to be unique, as a consequence of these protozoa possessing an unusual thiol metabolism. In these organisms, instead of glutathione, the major low molecular mass thiol is trypanothione [N^1^, N^8^-bis (glutathionyl) spermidine] [4], [5]. It has been recently reported that the GLOI system in *L. major* and *L. donovani* uses trypanothione as the substitute for glutathione [4], [5]. The metal cofactor is zinc in eukaryotes and nickel in *E. coli* [20], [21] and *L. major* [4]. Thus both the substrate and cofactor of *Leishmania* glyoxalase are different from the mammalian glyoxalases. The difference in cofactor dependence is reflected in differences between the active sites of the human and *Leishmania* enzymes, suggesting that the latter may be a target for antimicrobial therapy [22], [23].

Localization studies using *L. donovani* GLOI–GFP translational fusion shows that GLO I is localized not only in the cytosol of the parasites but also in the kinetoplast. Analysis of *L. donovani* GLOI sequences with MitoProt II [9] suggested targeting of proteins to the mitochondria. The GLO I enzyme is likely to be exported to the mitochondria with a *P* = 0.8780. Anlaysis of the *L. donovani* GLOI sequences with PSORT also suggested it to be a mitochondrial protein. This observation is supported from the sequence analysis of *T. cruzi* and *L. major* GLOI with MitoProt II having a P = 0.909 and 0.8704 respectively [24].

The biological function of a gene in an organism can be assessed by generating a knockout line and then characterizing the phenotype of the mutant. The functions of several genes of *Leishmania* have been established using gene targeting procedures [25], [26], [27], [28]. Our earlier studies have shown that the *GLOI* is a single copy gene and the expression is highest in late log phase promastigotes [5]. Homologous recombination strategy has been used to obtain single knock out mutants *Ld GloI :: HYG* (+/h) and *Ld GLOI :: NEO* mutants (+/n). Both these mutants exhibited slow growth rate and decrease in the specific activity of the glyoxalase I enzyme when compared to the wild-type. The decrease in enzymatic activity probably causes accumulation of MG derived adducts and induces oxidative stress in the mutants. Loss of one *GLOI* allele by gene disruption was compensated by genetic complementation with an episomal copy of GLOI. Our attempts to generate *GLOI* null mutants by step wise gene replacement failed despite the successful disruption of the two alleles by gene targeting. Earlier reports show that in *L. major*, generation of dihydrofolate reductase-thymidylate synthase null mutant [29] resulted in an increase in the chromosome number in order to keep one dhfr-ts allele intact. Similarly disruption of the trypanothione reductase (TR) gene of *Leishmania* led to partial trisomy for the *TR* locus [30]. It has been proposed that such alteration in the genome could be an indicator of the essential nature of the gene. In order to address this problem in the present study, the DNA content of the mutant lines was determined by FACS analysis and compared to that of the wild type. A normal diploid DNA content was observed in the wild type, heterozygous mutants with *hyg* replacement cassette (+/h), heterozygous mutant with *neo* replacement cassette (+/n), and GLOI complementation mutant (+/h-GLOI^+^). However, analysis of the DNA content of the double targeted transfectant (+/h/n) indicated that the second round of gene replacement resulted in aneuploidy. This change in ploidy has occurred during the introduction of the second replacement construct. The mechanism by which such changes in ploidy occur during gene replacement is not known, but this phenomenon is an indication that the gene being targeted for replacement is essential.

In order to test the hypothesis that the *GLOI* is an essential gene, an attempt was made to express the gene from an episome by previous transfection with a plasmid carrying the target gene for episomal expression and genetic complementation before the second round of gene replacement. We were successful in replacing both *GLOI* loci with two selection markers only in the presence of an episomal copy of GLOI in the heterozygous mutant. This shows that the *Leishmania donovani GLOI* gene is essential for the survival.

Difference in response to MG of the wild-type and the single allele knockout mutant further shows that mutants probably have a significantly reduced rate of metabolism of MG compared to the wild-type parasites. The heterozygous mutant *Ld GLOI :: HYG* (+/h) when complemented with an episomal copy of *GLOI* was able to overcome the effect caused by the deletion of one of the allele of *GLOI*. It has been implicated that glyoxalase I serves as an indicator of cell growth status in numerous cells, as its activity is low in resting cells and high during active cell growth and division [31], [32], [7]. The balance between methylglyoxal (the naturally occurring substrate of glyoxalase I) and glyoxalase I play a decisive role in the regulation of cell growth [33]. In the present study, it is possible that the single knock out mutants were unable to detoxify the cytotoxic effects of endogenous methyglyoxal and its accumulation induced cell growth retardation [34]. Similar observations have been made in the *GLOI* null mutants of *Escherichia coli* [35]. Inactivation of the *GLOI* gene sensitizes cells to MG. The phenotypic characteristics of the null mutants in the presence of an episomal copy of GLOI (−/−GLOI^+^) when studied were found to be similar to that of GLOI complementation mutants (data no shown). This data suggests that glyoxalase I may play an essential role in survival of this pathogenic organism and the major therapeutic perspective emerging from this study is that *Leishmania* GLOI may be further validated as an interesting target to develop specific chemotherapeutic inhibitors against the parasite.

## Materials and Methods

### Materials

Trypanothione disulphide was purchased from Bachem (Switzerland). [α^32^P]-dCTP (3000 Ci/mmol) was acquired from Amersham Biosciences, U.K. All restriction enzymes and DNA modifying enzymes were obtained from MBI Fermentas (Germany). The pX63-NEO and pX63-HYG, vectors that encompass the neomycin phosphotransferase (*neo*) and hygromcin phosphotransferase genes (*hyg*) respectively were kindly provided by Dr. Stephen M. Beverley (Washington University, St. Louis, MO). Construct pGEM-7zfαNeoα (containing neomycin phosphotransferase as the selection marker) with GFP was kindly provided by Dr. Marc Ouellette (University of Laval, Quebec, Canada). The antibody anti-F1 ATP synthase α-subunit (anti-mouse polyclonal serum) was kindly provided by Dr. Samit Adhya, Indian Institute of Chemical biology, Kolkata, India. Probes for *neo*, *phleo* and *hyg* were obtained by excision of pX63-NEO, pX63-PHLEO and pX63-HYG with *Spe*I. The DNA probe used in the present study included a 426-bp *GLOI* specific probe. Hygromycin B, Phleomycin and Geneticin (G418) were obtained from Sigma Aldrich Corp. (St. Louis, MO). Alexa-546 labeled IgG was obtained from Molecular Probes, Eugene, OR. The other materials used in this study were of the highest purity and were commercially available.

### Cell Culture

*L. donovani* promastigotes were grown at 22°C in M199 medium (Sigma St. Louis, MO) supplemented with 100 units/ml penicillin (Sigma, St. Louis, MO), 100 µg/ml streptomycin (Sigma, St. Louis, MO) and 10% fetal bovine serum (FBS, Hyclone, U.K.). All mutants of *GLOI* gene used in this study were derived from the wild-type *L. donovani* strain AG83 (MHOM/IN/1983/AG83) designated *GLOI* (+/+) for the purpose of genetic manipulations described here where *GLOI* denoted the Glyoxalase I locus and + specifies the wild-type allele. Promastigotes of mutant parasites containing functional copies of the neomycin or phleomycin resistant gene or hygromycin phosphotransferase gene were routinely maintained in the same medium supplemented with either 40 µg/ml Geneticin (G418)or 25 µg/ml phleomycin or 200 µg/ml hygromycin respectively. For characterizing the mutant parasites phenotypically, cells were subcultured without the selection marker prior to the experiments.

### Growth studies

Growth rate experiments were conducted by inoculating stationary phase parasites at a density of 1 ×10^6^ cells/ml in modified M199 medium with 10% FBS in 25 cm^2^ flasks without respective selection drug and cultured at 22°C. Growth rates of each of the cultures were determined at 24 h intervals by Neubauer haemocytometer. Growth studies with each individual cell line were done at least three times and similar results were obtained consistently.

### MTT assay

25 µl of 1× 10^6^ parasites/ml of mid log phase promastigotes were cultured in 96 well tissue culture plates and incubated with 25 µl of varying concentrations of methylglyoxal at 22°C. After 72 h, 20 µl of 5 mg/ml MTT [3-(4, 5- dimethylthiazol-2-yl) -2, 5- diphenyltetrazolium bromide] (Sigma) dissolved in PBSG (phosphate buffered saline with 1% of glucose, pH, 7.4) was added. Plates were incubated for 6 h at 37°C. Reaction was stopped by the addition of 50 µl of 50% isopropanol and 10% SDS by gentle shaking at 37°C for 30 min to 1 h. O.D. was measured spectrophotometrically at 570 nm [36]. Results are representative of three independent experiments.

### DNA extraction, Southern Blotting and Hybridization

Genomic DNA was extracted from *Leishmania* using a standard method [37]. 5 µg of DNA was digested with appropriate restriction enzymes, separated by 0.8% agarose gel electrophoresis, stained with ethidium bromide, transferred onto nylon membranes (Amersham Pharmacia Biotech) and subjected to Southern blot analysis. Blots were hybridized with denatured [α^32^P] -dCTP labelled DNA probe at 10^6^ c.p.m/ml, which was labelled by random priming (NEB Blot^®^Kit, New England Biolabs, Inc.) Membranes were washed, air dried and exposed to an imaging plate. The image was developed by Phosphoimager (Fuji FLA −5000) using ImageQuant software (Amersham Biosciences).

### Construction of GLOI-GFP fusion construct (pGEM-7zfαNeoα-GLOI-GFP)

The *GLOI* gene was amplified from the genomic DNA of *L. donovani,* using the primers 5′- CCCAAGCTTATGCCGTCTCGTCGTATG-3′ and 5′-CCCAAGCTTGGCAGTGCCCTGCTCCTT-3′ containing a flanking *Hin*dIII site (Underlined). The conditions for PCR were as follows: 94°C for 10 min, then 35 cycles of 94°C for 45 sec, 57°C for 45 sec and 72°C for 1 min. Final extension was carried out for 10 min at 72°C. The *GLOI* PCR product was digested with *Hin*dIII and cloned in to the vector pGEM-7zfαNeoα (containing neomycin phosphotransferase as the selection marker) with GFP at the carboxy terminal of the *GLOI* gene product. The clone was checked for correct orientation of the insert by restriction enzyme digestion and confirmed by sequencing. 20 µg of the plasmid, pGEM-7zfαNeoα*-GLOI-GFP* were transfected into the wild type *L. donovani*. Methods of electroporation and plating of *L. donovani* have been described previously [37]. The transfected cells were maintained in 40 µg/ml of G418. These transfected parasites were used for localization studies. A control transfection with vector alone, pGEM-7zfαNeoα-GFP was transfected in to the wild type *L. donovani* parasites for comparison.

### Localization of GLOI

To detect the site of localization of GLOI in *L. donovani*, pGEM-7zfαNeoα-*GLOI-GFP* transfected promastigotes were used. Fluorescent imaging of the stabilized culture was performed using the confocal laser scanning microscope (Zeiss LSM 510 META) equipped with a 63×objective, at an excitation wavelength of 488 nm. Briefly 10^7^ promastigotes/ml were pelleted and stained with MitoTracker Red –CMX-Ros (1 nM) (Molecular Probes, Eugene, OR) in a fresh media for 10–15 min to locate the kinetoplast with in the cell. The cells were then washed with phosphate-buffered saline (PBS) containing 1% fetal bovine serum (FBS) and resuspended in the same PBS solution with identical final cell concentration. The promastigotes were then immobilized on poly (L) lysine coated cover-slips. The cover-slips were incubated for 1 h at 37°C to allow parasite adherence. The cells were then treated with methanol/acetone (1∶1) for 15 min at −20°C followed by washing with PBS for 5 min. For observing the fluorescence of the parasites stained with Mitotracker Red –CMX-Ros the parasites were visualized under confocal laser scanning microscope at an excitation wavelength of 568 nm.

### Immunofluorescence Microscopy

10^6^ promastigotes/ml of the were pelleted and washed twice with phosphate- buffered saline (PBS) containing 1% fetal bovine serum (FBS) and resuspended in the same PBS solution with identical final cell concentration. The parasites were then immobilized and fixed as mentioned above and were permeabilised with PBS containing 0.5% Triton X-100 for 5 min, followed by washing with PBS. Blocking was done using PBS with 1%BSA. The cells were then incubated in a humidity chamber for 1 h at room temperature with mouse polyclonal anti-GLOI antibody (1∶100 dilution). After washing with PBS, cells were incubated with secondary anti-mouse Alexa-546 labeled IgG (Molecular Probes, Eugene, OR) (diluted 1∶2000) for 1 h. The cells were washed with PBS and incubated with DAPI (0.1 µg/ml) for 15 min at room temperature. The cover- slips were mounted on the slides and the parasites were visualized under the confocal laser scanning microscope at an excitation wavelength of 543 nm for immunoflourescence. The parasites stained with DAPI were observed at an excitation wavelength of 405 nm

### Cloning procedures and molecular constructs for the replacement of GLOI alleles by homologous replacement

Plasmid constructs pX63-HYG, pX63-NEO and pX63-PHLEO were used for the targeted replacement of *GLOI* allele in the wild-type *L. donovani* strain AG83. To construct drug resistance cassettes to replace *GLOI* alleles, 542-bp of 5′-flanking region and 1101-bp of 3′-flanking regions of *GLOI* gene were sub-cloned into the appropriate sites of the pX63-NEO, pX63-HYG and pX63-PHLEO plasmids. The 542-bp 5′- flanking region was amplified from the AG83 genomic DNA by PCR using a sense primer 5′ CCCAAGCTTACGGAATAGCGCGACTTG 3′ and the antisense primer 5′ ACGCGTCGACGAAAGGAGGAAGGGCAGA 3′, containing *Hin*dIII and *Sal*I sites (underlined) corresponding to a region ∼217 nucleotides upstream from *GLOI* ORF. The 1101-bp 3′ flanking region was also amplified from the AG83 genomic DNA by PCR using a sense primer, 5′CGCGGATCCCCTGTCTGCCTCGCTGAC 3′ and the antisense primer, 5′ GAAGATCTGCCGGATCAAGCGTTCTC 3′ containing *Bam*HI and *Bgl*II sites (underlined). The 3′ flank PCR product (∼1101-bp) was then sub-cloned into the *Bam*HI- *Bgl*II site of pX63-NEO, pX63-HYG and pX63-PHLEO. The resulting plasmids were termed pX63-NEO-3′F and pX63-HYG-3′F, pX63-PHLEO-3′F respectively. The 5′ flank PCR product (∼542-bp) was then sub-cloned into the *Hin*dIII- *Sal*I site of pX63-NEO-3′F, pX63-HYG-3′F and pX63-PHLEO-3′F to yield the three replacement constructs, pX63-NEO-Δ*GLOI*, pX63-HYG-Δ*GLOI* and pX63-PHLEO-Δ*GLOI.* respectively. The correct orientation of 5′ flank region and 3′ flank region within the three drug resistance cassettes was confirmed by automated sequencing and restriction mapping.

In order to generate heterozygous mutants of *GLOI*, five micrograms of the linearized DNA of the targeting constructs pX63-NEO-Δ*GLOI* or pX63-HYG-Δ*GLOI* (digested with *Hin*dIII and *Bgl*II) was transfected into mid log phase *L. donovani* Ag83 wild-type promastigotes by using standard electroporation conditions for transfection as reported earlier [37]. Transfection with linearised pX63-NEO-Δ*GLOI* or pX63-HYG-Δ*GLOI* into the wild type *L. donovani* was done to create the hetrozygote mutants, *L. donovani GLOI* :: NEO (+/n) and *L. donovani GLOI :: HYG* (+/h) respectively. Transfected *Leishmania* strains were maintained in liquid medium for at least 24 h before plating on a drug-containing semi-solid medium. After selection and stabilization of the liquid culture, mutant parasites were spread on the plates containing 1% Bacto- Agar (DIFCO) in M199 media (with 10% FBS) with either G418 (40 µg/ml) or hygromycin (200 µg/ml) as the case may be, to select for single colonies. The genotype of the *L. donovani GLOI* :: *NEO* (+/n) and *L. donovani GLOI* :: *HYG* (+/h) heterozygotes was confirmed by Southern blot analysis. A heterozygous clone of *L. donovani GLOI :: HYG* (+/h) was then subjected to a second round of transfection with linearised pX63-NEO-Δ*GLOI* to obtain null line Δ*GLOI*. These double targeted transfectants, *L. donovani GLOI :: HYG :: NEO* (+/h/n) were selected on semi-solid plates containing 1% Bacto- Agar (DIFCO) in M199 media (with 10% FBS) with both G418 (40 µg/ml) and hygromycin (200 µg/ml). A total of 10 clones of double targeted transfected line (+/h/n), were picked and screened by Southern blotting using *GLOI* ORF as a probe.

Selected clones from *L. donovani GLOI :: HYG* (+/h) mutants were also confirmed for the presence of the hygromycin integration cassette using the *hyg* specific internal forward primer, P2, 5′ -GACTG TCGGGCGTAC ACA- 3′corresponding to a region 905-bp downstream of the *hyg* start codon and a reverse primer, P1, 5′- GCAGCGATGGACACCAGAC- 3′ corresponding 7to a region 138-bp downstream from the 3′flanking region of *GLOI* ORF (Fig. 2). Selected clones from *L. donovani GLOI* :: *NEO* (+/n) mutants were checked for the presence of the neomycin integration cassette using the *neo* specific internal forward primer, P3, 5′-GGACCGCTATCAGGACAT- 3′corresponding to a region 151-bp downstream of the *neo* start codon and a reverse primer, P1, 5′- GCAGCGATGGACACCAGAC- *3*′corresponding to a region 138-bp downstream from the 3′flanking region of *GLOI* ORF (Fig. 2). The gene replacement of four of the double transfectant clones *L. donovani GLOI :: HYG :: NEO* (+/h/n) was further evaluated by polymerase chain reaction using primers P3 and P1 and also P2 and P1 (Fig. 2).

### Genetic complementation of the GLOI heterozygote mutant

*GLOI* gene was amplified using the primers 5′CCCAAGCTTATGCCGTCTCGTCGTATG 3′ and 5′CCCAAGCTTGGCAGTGCCCTGCTCCTT 3′ using a flanking *Hin*dIII site (Underlined). The conditions for PCR were as follows: 94°C for 10 min, then 35 cycles of 94°C for 45 sec, 57°C for 45 sec and 72°C for 1 min. Final extension was carried out for 10 min at 72°C. The *GLO I* PCR product was digested with *Hin*dIII and cloned in the vector pGEM-7zfαNeoα (containing neomycin phosphotransferase as the selection marker) to create pGEM-7zfαNeoα-LdGLOI containing the neomycin phosphotransferase gene. The representative heterozygous mutant, *L. donovani GLOI* :: *HYG* (+/h) were transfected with pGEM-7zfαNeoα -LdGLOI construct and transfected parasites were then selected in G418 (40 µg/ml) and hygromycin (200 µg/ml) in M199 media containing 10% FBS. Transfectant lines harboring episomes encompassing the wild-type and single deletion construct (linearized pX63-HYG-Δ*GLOI*) were designated as +/h–*GLOI* ^+^.

### Creating double mutants after episomal complementation of the GLOI heterozygote mutant

The transfectant cell line harboring the episomes encompassing the wild-type and single deletion construct (linearized pX63-HYG-Δ*GLOI*) designated as +/h–*GLOI* ^+^ was transfected with linearized pX63-PHLEO-Δ*GLOI* construct in an an attempt to create double mutants. These transfectants were selected on semi-solid plates containing 1% Bacto- Agar (DIFCO) in M199 media (with 10% FBS) with G418 (40 µg/ml), phleomycin (25 µg/ml) and hygromycin (200 µg/ml). A total of 9 clones were picked and screened by Southern blotting using *GLOI* probe. In order to check for the correct integration of *PHLEO* based replacement cassette *phleo* gene probe was used.

### Glyoxalase I activity

1×10^8^ promastigotes each from wild type *L. donovani*, *L. donovani* mutant cells were harvested in the late log phase by centrifugation at 1500×g, at 4°C for 15 min, washed with PBSG, pH-7.4. The activity of glyoxalase I was assayed spectrophotometrically at room temperature by measuring the initial rate of formation of S-D-lactoyl trypanothione at 240 nm as described by Racker with slight modification [38]. Trypanothione disulphide (1 mM) (Bachem) was reduced with 3 mM DTT at 60°C for 20 min before the assay. The resulting reduced trypanothione was used for GLOI assay. The assay mixture contained, in a final volume of 0.5 ml; 100 mM MOPS buffer, pH 7.2; 400 µM methylglyoxal (Sigma); 300 µM reduced trypanothione and 20 µM NiCl_2_ [4]. The assay mixture was incubated for 10 min followed by the addition of *Leishmania* cell lysate. Trypanothione hemithioacetal concentration was calculated by using the published K_d_ value of 3 mM for the methylglyoxal – glutathione equilibrium [39]. The value for the Δε_240 nm_ was taken as 2.86 mM^−1^ cm^−1^ for the isomerization of trypanothione hemithioacetal of methylglyoxal [40]. All assays were performed in triplicate.

### Western blot analysis

Late log phase promastigotes (1×10^8^) from GLOI-GFP transfectants were harvested and the resultant cell pellet was resuspended in lysis buffer (20 mM MOPS, pH 7.2; 1 mM DTT; 2 mM phenylmethylsulfonylfluoride (PMSF); 0.5 µg ml−1 each of leupeptin and aprotinin). The cell pellet was lysed by freeze thaw and cell supernatants were prepared by centrifugation at 20,000 × g. The supernatant was collected and was used as cytosolic fraction [41].

For crude mitochondrial fraction mid log phase promastigotes were harvested and washed with PBSG. The cell pellet was resuspended in 5 mM Tris, pH- 7.4 and kept at room temperature for 10 min. Suspension was homogenized using Potter Elvehjem Homogenizer and centrifuged at 3,500 rpm for 15 min to remove cellular debris. Supernatant was taken in eppendorf and centrifuged for 20 min at 12,000 rpm. Pellet was resuspended in phosphate buffer (5 mM Na-PO_4_), pH-7.4 at a protein concentration of 10 mg/ml [42]. All centrifugation steps and other operations were performed at 4°C. 100 µg of protein from each fraction was fractionated by SDS-polyacrylamide gel electrophoresis, blotted on to PVDF membrane using electrophoretic transfer cell (Bio-Rad) and probed with GLOI specific polyclonal antibody raised in mice (diluted 1∶500) [5]. The blots were developed with 3, 3′-diaminobenzidine (Sigma).The mitochondrial and cytosolic fractions were also probed with mitochondrial specific anti –F1 ATP synthase α- subunit (anti–mouse polyclonal serum; diluted 1∶250) [43].

### DNA Content Analysis

*L. donovani* promastigotes (1 ×10^7^ cells/ml) were harvested by centrifugation at 2000 × g for 5 min at 4°C. Cells were washed once in phosphate-buffered saline (PBS; 0.01 M phosphate buffer, 2.7 M KCl, 0.137 M NaCl, pH-7.4). The cells were then fixed by incubation in 70% methanol/30% PBS for 1 h at 4°C. Prior to analysis fixed cells were harvested by centrifugation at 1000 × g, for 10 min at 4°C, washed in PBS and then resuspended in 1 ml PBS with RNAse A (10 µg/ml) and incubated at 37°C for 20 min. Following this propidium iodide (50 µg/ml) was added. Samples were then analyzed using a flow cytometer (FACS, Beckton Dickinson). For each sample at least 10,000 cells were analyzed.
